# Health Among Sexual Minority Older Adults: Individual Differences and Implications for Improving Health

**DOI:** 10.1093/geroni/igaa057.3170

**Published:** 2020-12-16

**Authors:** Britney Wardecker, Cara Exten

## Abstract

The number of sexual minority (SM) older adults is increasing rapidly, yet this population continues to be underrepresented in research (Fredriksen-Goldsen & Kim, 2017) and experiences significant disparities in health and health care access (Fredriksen-Goldsen, 2016; Wallace et al., 2011). In the current symposium, we analyze data from U.S. national probability samples of middle-aged and older adults (MIDUS, HRS, NESARC-III) to consider how age-related concerns and challenges may be experienced differently by SM individuals compared to their heterosexual counterparts. This symposium includes novel methods and statistical tools, such as daily diary assessments, multilevel modeling, and time-varying effects models. Individual presentations evaluate how: (1) SM women, compared to heterosexual women, may respond differently to menopause through norms and values surrounding womanhood; (2) midlife and older SM individuals use alcohol and cigarettes more frequently across a typical week than their heterosexual counterparts, though their substance use may not be tied to common triggers (e.g., negative mood, stress); (3) despite bisexual older adults reporting more health problems compared to lesbian and gay counterparts, they are less prepared for health concerns and crises (e.g., reporting a lower number of valid wills); and (4) the prevalence of depression and anxiety varies across age, such that older SM adults—especially women—are particularly vulnerable to psychological health problems. These presentations collectively examine complex issues facing older SM adults while emphasizing individual differences (i.e., women’s concerns, bisexual people’s issues). We discuss challenges in researching this growing at-risk population, and we highlight areas of future research and intervention.

